# Electroencephalogram (EEG) dataset with porn addiction and healthy teenagers under rest and executive function task

**DOI:** 10.1016/j.dib.2021.107467

**Published:** 2021-10-09

**Authors:** Xiaoxi Kang, I Made Artha Agastya, Dini Oktarina Dwi Handayani, Mun Hou Kit, Abdul Wahab Bin Abdul Rahman

**Affiliations:** a School of Computer Science and Engineering, Faculty of Innovation and Technology, Taylor's University, 47500 Subang Jaya, Selangor, Malaysia; b Department of Informatics, Universitas Amikom Yogyakarta, Jl. Ringroad Utara, Condong Catur Depok, Sleman Yogyakarta 55583, Indonesia; c Department of Mechatronic and Biomedical Engineering, Faculty of Engineering and Science, Universiti Tunku Abdul Rahman, Bandar Sungai Long, Selangor 43000, Malaysia; d Department of Computer Science, Faculty of Information Communication and Technology, International Islamic University Malaysia, 50728 Gombak, Kuala Lumpur, Malaysia

**Keywords:** Electroencephalogram, Emotional state, Teenager pornography addiction, Working memory capacity

## Abstract

The electroencephalogram (EEG) signal data were obtained from Yayasan Kita dan Buah Hati (YKBH), Jakarta, Indonesia and collected using a Brain Maker EEG machine with 19 channels. The sampling rate of the machine was 250 Hz. Fourteen participants (five females and nine males) participated in the data collection. A psychologist verified that seven of them were addicted to porn, and seven were healthy teenagers. The EEG data were recorded using one protocol with nine tasks for 10 min. The three stages were the baseline (tasks with eyes closed and open), emotional state (happy, calm, sad and fearful tasks) and main (15-words memorisation task, executive task and 15-words recall task) stages. The data obtained was used to analyse the signal pattern of pornography addiction amongst teenagers, as well as the emotional signal pattern and working memory capacity.

**Specifications Table**

**Table:** 

Subject	Neuroscience
Specific subject area	Cognitive neuroscience, electroencephalogram (EEG) signal, porn-addicted brain activity, emotions, working memory capacity
Type of data	Electroencephalography data
Mode of data acquisition	Data was acquired using a Brain Maker EEG machine with 19 channels. The sampling rate of the device is 250 Hz.
Data format	Raw data (.csv)
Parameters for data collection	A total of 14 teenagers (five female and nine male) between 13 and 15 years old participated in the study. Using interviews and questionnaires, a clinical psychologist verified seven of them as porn-addicted and seven as healthy teenagers.
Description of data collection	The EEG data were recorded through one of the protocols in 10 min. There were three stages in the protocol: baseline stage comprising tasks with eyes in the closed and open state, emotional stage (happy, calm, sad and fear) while watching the International Affective Picture System (IAPS) [1] image tasks, and main stage consisting of 15-words memory task, watching pornographic images, and recalling 15 words task.
Data source location	Taylor's University 1, Jalan Taylors, 47500 Subang Jaya, Selangor, Malaysia
Data accessibility	Link: https://data.mendeley.com/datasets/4r8hp2hmb4/5 DOI: 10.17632/4r8hp2hmb4.5

**Value of the Data**

• The dataset is useful for analysing the brain activity in pornography addiction cases through electroencephalogram signals.
• The dataset is useful for analysing emotions amongst teenagers through EEG signals.
• The dataset is useful to analyse the working memory capacity amongst teenagers through EEG signals.
• The dataset is beneficial for data scientists and psychologists to understand pornography addiction amongst teenagers in detail.
• The dataset can be used on different frequency bands and other brain regions to understand the frequency band or the particular brain area that shows the pattern of pornography addiction behaviour.

## 1 Data Description

This data in “Brief article” provided detailed information of the electroencephalogram (EEG) dataset with porn addiction and healthy teenagers under rest and executive function tasks [2]. We acquired EEG signal data from Yayasan Kita Dan Buah Hati (YKBH), Jakarta, Indonesia. Data were collected from 14 participants (five females and nine males). Seven of them were confirmed to be addicted, whereas seven were healthy youngsters, based on clinical evaluation by the psychologist. The detailed information on the stimuli, subject details, gender, label, and screening method is presented in the Experimental Design, Materials and Methods section.

**Fig. 1:**
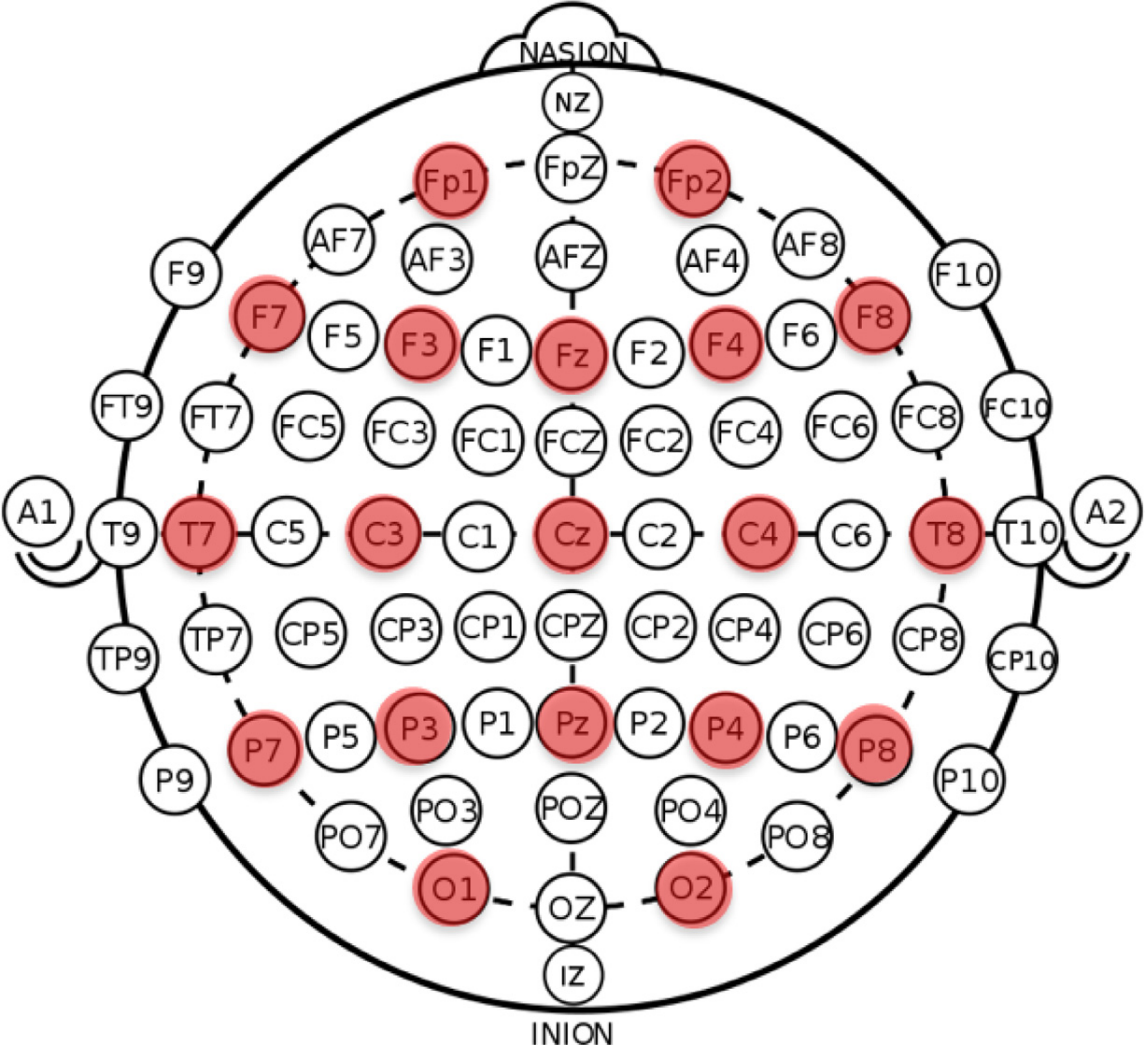
Placement of electrodes on the scalp highlighted with red colour.

The dataset was collected using a Brain Maker device with 19 channels. The channels are P4, O2, P8, T8, C4, Cz, Fz, F4, Fp2, F8, Fp1, F7, F3, C3, T7, P7, P3, O1 and Pz. Each participant had nine different tasks in the raw data, which included eyes closed and open, happy, calm, sad, fear, memorise, executive task and recall with a total of 10 min. EEG signals were acquired using the Brain Maker with 19 channels. The EEG electrodes were placed on the head, which followed the International 10–20 system, as shown in Fig. 1. The sampling frequency of the recorded data was 250 Hz. The EEG data of each participant was stored in a .tdms file. The software QtiPlot (https://www.qtiplot.com/download.html) was used to convert the .tdms file into .csv file.

Table 1 shows the detailed information on raw data, including the task and file names, duration, and array shape. The data array shape was arranged in samples × channels. The length of the data was manually validated by multiplying the sampling frequency with the task duration to obtain samples numbers for each task. For example, the number of samples in the closed-eye task was 250 Hz × 60 s = 15,000. Each sample had 19 channel signal values in microvolts.

**Table 1:** The EEG signals recorded for each subject.

No.	Task Name	File Name	Durations (seconds)	samples × channels
1	Eyes Closed	EC.csv	60	15,000 × 19
2	Eyes Open	EO.csv	60	15,000 × 19
3	Happy	H.csv	60	15,000 × 19
4	Calm	C.csv	60	15,000 × 19
5	Sad	S.csv	60	15,000 × 19
6	Fear	F.csv	60	15,000 × 19
7	Memorise 15 words	M.csv	60	15,000 × 19
8	Executive Tasks	ET.csv	120	30,000 × 19
9	Recall 15 words	R.csv	60	15,000 × 19

**Fig. 2:**
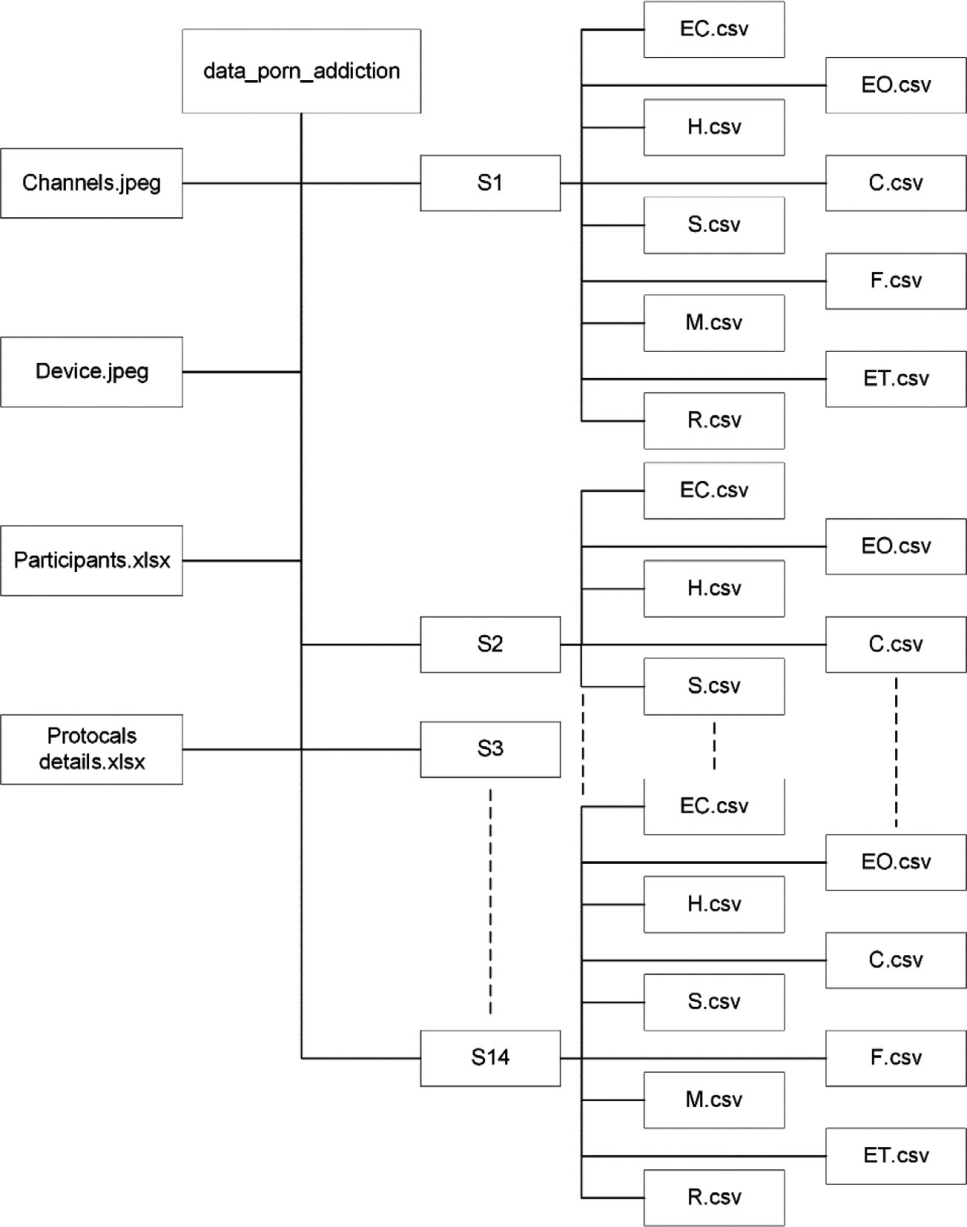
Dataset folder structure.

As shown in Fig. 2, the dataset was stored in a folder named data_porn_addiction. The data_porn_addiction comprised 14 folders that represented the subjects’ data, named S1 until S14. Each subject's folder contained several .csv files, such as EC.csv, EO.csv, H.csv, C.csv, S.csv, F.csv, M.csv, ET.csv and R.csv. We also provided detailed information such as channel representation in Channels.jpeg, data acquisition hardware in Device.jpeg, participant labels in Participants.xlsx, and experimental protocols in Protocols_details.xlsx.

## 2 Experimental Design, Materials and Methods

### 2.1 Stimuli

In this experiment, we used two stimuli. The first stimulus is the emotional state of the IAPS [1]. The stimulus showed images related to emotions, including happy, calm, sad and fearful. Image of each emotion was displayed for 1 min. Another stimulus was the executive task, showing pornographic images. The images were provided by a psychologist. We chose both stimuli because we wanted to know the brain activity of porn-addicted and non-porn-addicted subjects. Based on EEG signals collected in several emotional states using IAPS's standard procedure, we inferred the emotional state of the subjects during the executive task.

### 2.2 Subject

The data collection process included 14 participants with ages varying between 13 and 15 years. Table 2 presents the participants’ details. The psychologist verified that out of 14 subjects, seven were porn-addicted, while the rest seven did not have porn addiction using the Youth Pornography Addiction Screening Tool (YPAST) [3,4], which is a free on-line initial screening assessment tool for adolescents aged 12 to 18 years with potential addiction to pornography. The subjects answered each of the 25 questions by choosing one of the five possible responses. Moreover, the psychologist interviewed the participants and their parents to assess the YPAST results. By analysing both instrument results, the psychologist concluded the participant label or category.

**Table 2:** Participants details.

No	Subject ID	Sex	Label
1	S1	M	Addicted
2	S2	F	Not Addicted
3	S3	F	Not Addicted
4	S4	M	Not Addicted
5	S5	M	Addicted
6	S6	M	Addicted
7	S7	M	Not Addicted
8	S8	M	Not Addicted
9	S9	F	Addicted
10	S10	F	Addicted
11	S11	F	Addicted
12	S12	M	Not Addicted
13	S13	M	Not Addicted
14	S14	M	Addicted

**Fig. 3:**
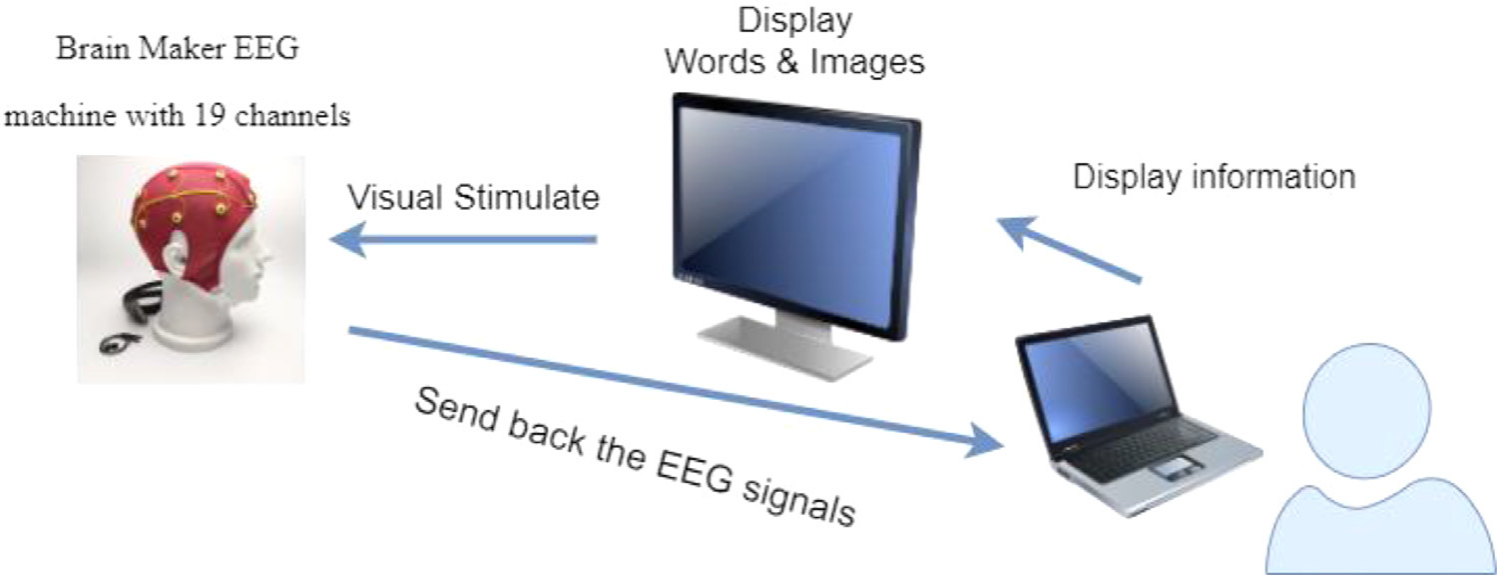
The experimental set-up.

### 2.3 Protocols

**Fig. 4:**
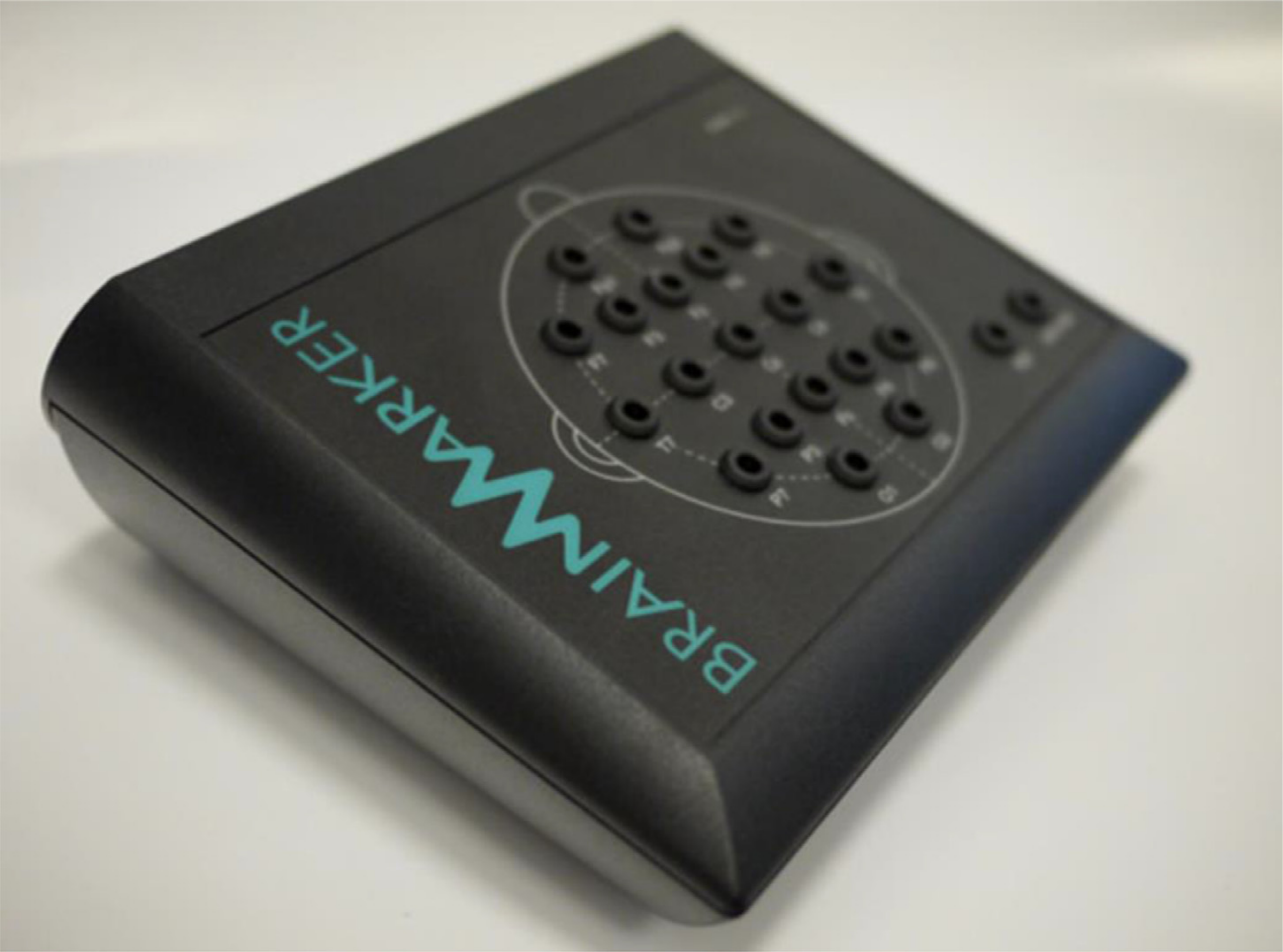
Brain Maker device with 19-channel electrodes.

As shown in Fig. 3, the participant watched the visual stimulus from the monitor by wearing the Brain Maker device. The protocol for controlling the visual stimulus by an administrator is presented in Table 3. Signals were collected when the participant was performing the task. Fig. 4 shows the Brain Maker device used in this experiment.

**Table 3:** Overview of the protocols.

Duration	Stage	Task	
2 min	Baseline		Eyes Closed (1 min)

	Eyes Open (1 min)

4 min	Emotional State		Happy (1 min)

			Calm (1 min)

	Sad (1 min)

	Fear (1 min)

4 min	Main	Memorize 15 words (1 min)	
		Executive Tasks (2 min)	
		Recall 15 words (1 min)

The three experimental stages comprised a total of nine tasks. The experiment duration for each participant was 10 min. Table 3 shows the task names and duration for each stage. The three stages included the baseline, emotional state and main stages. The baseline stage consisted of tasks with eyes open and eyes closed. In the emotional state stage, each of the happy, calm, sad and fearful states were collected for 1 min with IAPS [1] as the stimulus. It provided a set of coloured photographs of the normative emotional stimulus. The monitor showed a picture to elicit a happy, calm, sad or fearful emotion in the participants. In the “Memorise 15 words” task, the participant watched 15 words displayed individually on the monitor for 1 min. Each participant was instructed to remember all displayed words. In the executive task, the participant watched a series of images containing pornography items validated by a psychologist, for 2 min. In the recall task, the participants were asked to spell the 15 words given in the “Memorize 15 words” task in 1 min while watching a white blank screen.

## Ethics Statement

This study was approved by Taylor's University (HEC 2020/035). The ethics committee approved all questionnaires and tasks for the participants. The parents and legal tutors of the children received written information about this research and a consent form. The participants and their legal tutors read and signed the consent form before the experiment. The EEG device on the participant's head did not cause any pains or injections or both. Consequently, the participants completed the experiment safely. The study was conducted according to the guidelines of the Code of Ethics of the World Medical Association (Declaration of Helsinki) and approved by the Ethical Committee Taylor's University Malaysia

## CRediT authorship contribution statement

**Xiaoxi Kang:** Formal analysis, Writing – original draft, Writing – review & editing. **I Made Artha Agastya:** Formal analysis, Writing – review & editing. **Dini Oktarina Dwi Handayani:** Data curation, Investigation, Validation. **Mun Hou Kit:** Supervision. **Abdul Wahab Bin Abdul Rahman:** Supervision.

## Declaration of Competing Interest

The authors declare that they have no known competing financial interests or personal relationships which could influence the work reported in this article..
